# Use of computerized tests to assess the cognitive impact of interventions in the elderly

**DOI:** 10.1590/S1980-57642014DN82000004

**Published:** 2014

**Authors:** Rafaela Sanches de Oliveira, Beatriz Maria Trezza, Alexandre Leopold Busse, Wilson Jacob Filho

**Affiliations:** 1 Hospital das Clínicas da Faculdade de Medicina da Universidade de São Paulo – USP/SP, São Paulo SP – Brazil.

**Keywords:** elderly, neuropsychological tests, diagnosis by computer, intervention studies

## Abstract

With the aging of the population, the possibility of the occurrence of cognitive decline rises. A number of types of intervention seek to attenuate or reverse this impairment. The use of computerized tests helps quantify the effects of interventions on cognitive function in the elderly. The objective of the present review was to analyze studies that have utilized computerized cognitive tests to determine the effects of interventions in the elderly population, describing the batteries and tests employed, the populations studied and reports by authors on the limitations or benefits of employing these tests in older adults. The review was performed on the PubMed database using the descriptors: cognitive computerized test and elderly. We retrieved 530 studies and, following analysis of their abstracts, selected 32 relevant to the subject. The studies utilized 19 different types of computerized tests and batteries to assess the interventions, which were predominantly drug trials. There were no reports on limitations in the use of the computerized tests, suggesting this type of intervention had good applicability, sensitivity, and little or no practice effects in this population.

## Abstract (pt)

Com o envelhecimento da população aumenta a possibilidade de ocorrência de um declínio cognitivo. Diversas formas de intervenção buscam amenizar ou reverter este prejuízo. O emprego de testes computadorizados auxilia na detecção dos efeitos das intervenções nas funções cognitivas dos idosos. O objetivo desta revisão foi o de analisar os estudos que utilizaram testes cognitivos computadorizados para verificar o efeito de intervenções na população idosa, descrevendo as baterias e testes empregados, as populações analisadas e o relato dos autores sobre limitações ou facilidades do emprego destes testes na população. Foi revista a base de dados PubMed com os descritores: teste cognitivo computadorizado e idoso. Encontramos 530 estudos e, pela leitura dos resumos, selecionamos 32 pela relevância com o tema. Estes utilizaram 19 tipos de testes ou baterias computadorizadas para avaliar as intervenções, que na sua maioria foram medicamentosas. Não houve relato de limitações na utilização dos testes computadorizados, sugerindo que a aplicação desta forma de avaliação tenha boa aplicabilidade, sensibilidade e pouco ou nenhum efeito de aprendizagem nesta população.

## INTRODUCTION

The rise in incidence of dementias is set to become the greatest public health concern in the coming decades. Early diagnosis does not yet translate to successful cure, but does allow the application of interventions to delay the evolution of the symptomatology and thereby maintain patient quality of life for a longer period. Toward this goal, the selection of tests that can be applied in the elderly population, offering practical repetition and reliability, is of increasing importance.^1^

The first studies on the effectiveness of computerized cognitive tests for diagnosing dementias and other cognitive changes date back to the 1980s. The studies demonstrated the benefits and limitations of these novel computer-based testing over conventional paper-and-pencil format tests. Current studies on the theme are aimed at developing more effective batteries with greater applicability.^2^

Computerized cognitive batteries are more accessible in terms of cost and in their need for operator training (often essential in self-applied batteries) and able to assess multiple cognitive functions, allowing standardization of assessments, greater consistency and global sensitivity, accuracy in detecting response speed, and can produce reports automatically. Given the highly accurate data recorded, computerized batteries have gone beyond use merely for epidemiological purposes, since they can aid comparison of performance at different timepoints in the same individual, under a variety of situations and between different individuals.^1,3^

However, a number of questions remain regarding the use of this assessment approach. Concerns include previous experience with using computers, known to be lesser in the elderly population; the anxiety created by the use of a computer during testing may compromise performance and the answers given, particularly when the results between repetitions are compared, since anxiety tends to abate with increased familiarity; the repetition of the tests within short time intervals may cause a practice/learning effect and lead to a false improvement in performance.^4^

The aim of this study was to review those studies which applied computerized cognitive batteries to verify the results of interventions in the elderly population, and thereby determine whether lack of experience in the use of technology, together with the anxiety generated during application of the tests, can actually compromise their applicability in the elderly population.

## METHODS

This study was performed on the Pubmed database in February 2014 with the following search descriptors: *cognitive computerized test* and *elderly*. A total of 530 abstracts were identified and analyzed. Only those articles involving elderly populations with a mean age of 60 years or older and computerized cognitive tests in the assessment of outcomes of interventions in this population were selected. A total of 32 articles were selected, only one of which was Brazilian.

Two further bibliographic review articles were included, on the use of computerized cognitive tests in the elderly, which helped us glean a better understanding of the batteries reported in the other studies reviewed.^2,5^

## RESULTS

Many of the 530 abstracts available on the database retrieved using the search descriptors did not directly address the elderly population. Numerous studies, despite having titles indicating the population analyzed was elderly, were later found to involve subjects whose mean age or age band was less than 60 years of age. Some studies claimed to involve elderly on the basis of containing only a few individuals in the over sixties age group among a much broader casuistic. Another factor leading to the exclusion of many studies in the present analysis was the interpretation of computerized tests, such as computed tomography, as being cognitive tests.

Given the objective was to analyze studies assessing the results of interventions in the elderly population through the application of computerized cognitive tests, epidemiological and test battery validation studies were not included in the present analysis.

The sample sizes of the studies analyzed showed that the majority of the tests were applied in a small number of individuals. Some 25% of the studies involved a sample size of under 20 subjects, 34.4% had 21-50 participants, while only 4 studies^12,17,28,29^ (12.5%) included over 100 individuals, one of which was cross-sectional 28 and the remaining three assessed response to medications in patients with Alzheimer's disease or memory impairment.^12,17,29^ We believe that the small casuistics may be due to reservations that this diagnostic technology may not be suitable for assessing the elderly population. The authors whose work involved small samples cited this reason as a limitation in their studies,^10,18,31-34^ suggesting that future studies be conducted in a larger number of individuals to confirm the results observed.

Regarding the profile of the casuistics, 84.4% (27) of the studies included both genders; 37.5% (12) involved elderly from the community; 18.7% (6) presented memory impairment; 12.5 (4) were diagnosed with Alzheimer's disease, 18.7% (6) were post-surgical patients; whereas patients with mild cognitive impairment, strokes, submitted to chemotherapy treatment, and residents of long-stay care facilities were each assessed by one study (3.2%).

Mean age of participants in the studies selected was approximately 68 years. This average was calculated based on the 31 studies which reported mean ages. In one study,^33^ the authors opted to express age of the study population stratified by age group from 61 to 78 years.

Level of schooling and cognition were also expressed in different ways. A number of authors did not report the educational level of the population studied^9,16,18,24,32,33,37^ while the majority expressed schooling as mean years of education^6,10,13,15,20,27,28,30,36^ giving an overall mean of 11.5 years and range of 14.1-4.2 years, with the latter lower figure attributed to the study conducted in Brazil. Among the studies opting to express schooling by category, a wide variety of classifications was observed. The interval for years of schooling varied, such as the study of Bozoki et al. (2013), in which 25% of the volunteers had between 13 and 15 years of education^7^ and the study by Cremer et al. (2011), in which 74.2% had schooling of 12 years or more.^14^ The level of educational attainment was another method of categorizing schooling. In a study carried out by Nagamatsu et al. (2013), 52.3% of those assessed had completed secondary or higher level education.^8^ Galvin et al. (2008) reported that 74.2% of elderly had completed higher education^22^ while the greatest schooling was reported by the study of Dunbar et al. (2007) in which 92% had concluded secondary or higher education.^25^

Some authors opted to characterize cognitive function by maximum and minimum scores on the Mini-Mental State Examination (MMSE), revealing an overall mean of 25.5 points, a minimum of 20 and maximum of 29.7.^19,26,30,34^ Other authors established a minimum value as a cut-off score (24 points).^12,17,21,31^ To a lesser extent, cognition was also expressed by Intelligence Quotient (IQ) score.^11,35^

The wide variation seen in MMSE scores and IQ tests may be due to the different populations tested, such as elderly from the community and Alzheimer's disease patients, and also the different objectives of these studies.

Schooling level also varied, although most studies reported educational levels, on average, of over eight years, double that of the level found in the Brazilian study,^28^ suggesting this more highly educated contingent had greater ease in understanding and carrying out the tests.

Two of the studies were cross-sectional,^28,36^ while the others repeated the cognitive tests two to five times, with an average of 2.9 repetitions. The time intervals between tests varied from several hours,^14,32^ to six years.^34^

Also with regard to the methodologies applied, four studies were prospective, 20 included a control group, of which 15 involved placebo. Only one study reported the use of paired samples,^26^ whereas 12 were randomized. Five studies were blinded and 20 double-blind.

The different methodologies applied varied mainly according to the type of intervention undertaken. Effects of medications were tested in 18 studies, with others addressing interference of surgery^9,27,29,36^, vitamins,^15,35^ hormones^24^ and other substances,^11^ transcranial magnetic stimulation,^19^ chemotherpay,^36^ anesthesia,^14^ cognitive training^7^ and physical training^8^ and activities in long-stay care facilities.^28^ The medications tested, in most cases, were used for the treatment of Alzheimer's disease, or as a means of improving cognitive performance in elderly with memory impairments.

A number of different batteries were used in the studies selected, some validated and others consisting of conventional tests adapted to computer-based versions. Several of the studies used tests developed by the authors, with all tests outlined in Table 1.

**Table 1:** Cognitive batteries and tests employed in studies reviewed.

Cognitive batteries and tests	Cognitive functions	Studies
Cognitive Drug Research - CDR**	Attention, working memory, episodic memory	^12, 16-18, 22, 23, 25, 29, 32, 34, 37^
Cog State™**	Reaction time, attention, executive functions, working memory, learning	^7, 30^
Groton Maze Learning Test - GMLT (part of Cog State™)*	Visual memory, attention, executive functions	^20,21^
Testing Attentional Performance - TAP*	Reaction time, attention, executive functions	^9, 14^
Cambridge Neuropsychological Test Automated Battery - CANTAB**	Planning, visuo-spatial memory, working memory, attention	^10, 33^
Computerized Neuropsychological Test Battery - CNTB**	Reaction time, executive functions, visual memory, episodic memory, learning, language	^12, 19^
Conners Continuous Performance Test - CPT	Attention, reaction time, executive functions	^13, 31^
Swinburne University Computerized Cognitive Assessment Battery - SUCCAB**	Reaction time, episodic memory, working memory, reaction time, attention	^15^
Seoul Computerized Neuropsychological Test **	Verbal memory, visual memory, working memory, executive functions, attention	^19^
CNS Vital Signs**	Executive functions, reaction time, visual memory, verbal memory and working memory	^11^
Nex Sig Neurological Examination Technologies - Nex Ade™**	Reaction time, attention, episodic memory	^6^
Neurobehavioral Examination System - 2 (NES-2)**	Attention, visual memory, episodic memory, executive functions	^24^
Psychologix Computerized Cogscreen Test Battery**	Episodic memory, visual memory, reaction time, learning, executive functions	^26^
Computer-administered Perceptual Matching - PM and Associative memory - AM*	Associative memory, reaction time	^27^
Hooper´s Test (computerized version)*	Visual memory	^28^
Automated Neuropsychologic Assessment Metrics - ANAM*	Reaction time, executive functions, and spatial memory	^31^
Sperling Whole Report Task *	Verbal memory, visual memory, episodic memory, attention and reaction time	^35^
Microcog**	Spatial memory, reaction time	^36^
Computerized Memory Battery Test - CMBT**	Episodic memory, verbal memory	^29^
Computerized test of spatial memory and reaction time*	Attention, spatial memory, working memory, reaction time	^8^

* Tests;

** Batteries.

For the application of the tests, computers, notebooks or tablets were used with user input via keyboard, response box or touchscreen function. Among the studies reporting duration of the tests, execution times ranged from 5^9,14^ to 90^10,33^ minutes. This wide variability in test time was due to the fact that some batteries contained a large number of tests, while for others completion time depended on the performance of the individual under assessment, advancing to levels of increased difficulty upon good performance (prolonging the test) or halting the test following a series of errors (shortening test time).

We found 15 studies that, although made use of computerized tests, did not dispense with conventional paper-and-pencil based tests. The studies citing the rationale for using the tests sought to compare their sensitivity and reliability,^12,14,30,36^ and to minimize and/or verify the practice effect.^14,19,20,30^ The conventional tests used included: the Rey Auditory Verbal Learning Test – RAVLT,^6,8,26,33^ and Alzheimer's Disease Assessment Scale - Cognitive – ADAS-Cog,^10,12,29^ with these being the most frequently employed. MMSE^28,29^ and Clinical Dementia Rating – CDR,^10^ often featuring in the selection of volunteers, were also used as instruments for assessing the evolution of patients. The Stroop test, Digit Symbol Substitution Test – DSST,^13,30^ and the Trail Making A and B,^14,24,30^ were applied in both their conventional and computerized versions in some batteries.

Regarding the performance exhibited by the elderly on the computerized cognitive tests, Pietrzak et al. (2009) stated that the Groton Maze Learning Test – GMLT showed good sensitivity and no learning effect in the placebo group. A reduced learning effect was also reported in the study of Cremer^14^ in the application of the Testing Attentional Performance. Akin to the study by Silbert,^30^ superior reliability and sensitivity besides a lower learning effect was found for the Cog State computerized battery as compared to the conventional tests: Word Learning Test, Symbol Digit Modalities Test, Trail Making A and B, Semantic Verbal Fluency and Grooved Pegboard Test.

In a more recent study by Oliveira,^38^ investigating the applicability of computerized cognitive tests and the possibility of learning upon repetition, showed that the tests were well accepted by the elderly population, with no difficulties in application and no learning effects, even after immediate repetition.

Out of the articles reviewed, the only study questioning the efficacy of the tests was the investigation that assessed executive functions and working memory before and after transcranial stimulation, using the Seoul Computerized Neuropsychological instrument. This conclusion was likely due to the absence of difference found after application of the intervention in executive functions, attributed by the authors to election of the wrong test for detecting them.

Given that most authors did not describe performance on the computerized tests or report difficulties encountered in their use, it can be assumed they were deemed easy to apply and understand by the elderly population, effectively fulfilling the purpose for which they were chosen.

The reviews on computerized cognitive tests in the elderly population published in recent years have described the batteries employed in this population, citing some tests used in the studies that served as a basis for the present study.^2,5^ Some of the batteries mentioned were not selected by us because the studies applying them were not aimed at detecting changes promoted by interventions.

These reviews were instead centered on validated cognitive batteries with a broad spectrum of assessment. The present study also included the experiences in applying simple tests assessing only a small number of cognitive functions, adapted versions of conventional tests, and even tests devised by the authors. We chose to include a wide variety of tests applied in elderly populations so as to broaden the scope of assessment settings. Nevertheless, no negative results regarding the use of computerized cognitive tests were found.

In conclusion, review of the 32 articles selected for this study revealed the use of 19 different types of computerized tests or batteries, allowing assessment of the impact of interventions, predominantly drug trials, applied in elderly individuals diagnosed with Alzheimer's disease or memory impairment.

There were no reports on limitations in the use of the computerized tests, implying that the application of this type of intervention had good applicability, sensitivity and little or no learning effect in this population.
